# Investigating the effect of rhubarb (*Rheum ribes* L.) root and leaf extracts on seed germination timing and early growth parameters: An approach to plant allelopathy

**DOI:** 10.1016/j.bbrep.2026.102496

**Published:** 2026-02-11

**Authors:** Saeed Sheikh Ali Babaei Mahani, Mohsen Sharafatmandrad, Esfandiar Jahantab

**Affiliations:** a Department of Ecological Engineering, Faculty of Natural Resources, University of Jiroft, Jiroft, Iran; b Department of Range and Watershed Management (Nature Engineering), Faculty of Agriculture, Fasa University, Fasa, Iran

**Keywords:** Allelopathy, *Rheum ribes*, Germination timing, Rangeland restoration, Species-specific response

## Abstract

This study evaluated the allelopathic effects of aqueous leaf and root extracts of *Rheum ribes* L. on two native rangeland species, *Stipa barbata* Desf. (Poaceae) and *Astragalus cyclophyllon* G. Beck (Fabaceae), focusing on both germination timing and early seedling growth. Extracts at 50% and 100% concentrations were applied in a controlled factorial experiment with four replications. Germination metrics (T_10_, T_50_, T_90_, mean germination time, and germination rate index) and seedling traits (epicotyl and radicle length and dry biomass) were measured. Statistical analyses (including ANOVA, Kaplan-Meier survival curves, and Cox proportional hazards models) showed that extract effects were strongly dependent on species, plant part, and concentration. *S. barbata* exhibited delayed germination and reduced root and shoot growth under concentrated root extracts, indicating high sensitivity. In contrast, *A. cyclophyllon* displayed variable responses, including radicle stimulation at some concentrations, alongside consistent suppression of epicotyl development. These species-specific and dose-dependent effects demonstrate that *R. ribes* releases bioactive compounds capable of altering germination dynamics and early seedling performance. The findings highlight ecological implications for plant-plant interactions and provide guidance for restoration efforts in *R. ribes*-dominated rangelands.

## Highlights

• Aqueous leaf and root extracts of *Rheum ribes* affected seed germination and early seedling growth.
• Germination timing and seedling traits responded in a species-specific and concentration-dependent manner.
• *Stipa barbata* showed delayed germination and strong growth inhibition, particularly under root extracts.
• *Astragalus cyclophyllon* exhibited variable responses, including radicle stimulation at certain concentrations.
• The allelopathic effects of *R. ribes* indicated potential implications for rangeland restoration planning.

## 1 Introduction

Rangelands are among the foundational components of natural ecosystems, providing essential ecological, economic, and social services that support biodiversity and sustain the livelihoods of pastoral communities [1]. They play a vital role in maintaining biological resources by supplying forage, preserving soil structure, recharging groundwater, and contributing to climate regulation at local and regional scales [2,3]. Despite their importance, rangelands have experienced increasing degradation over recent decades as a result of excessive grazing pressure, recurrent drought, land-use change, and the accelerating impacts of climate change [[4], [5], [6]]. Effective restoration therefore requires strategies that address abiotic limitations while also accounting for species-specific traits and plant-plant interactions that influence community dynamics [7,8].

One ecological mechanism strongly shaping plant coexistence and community assembly in rangelands is allelopathy: the release of bioactive secondary metabolites by plants that inhibit or stimulate the growth and development of neighboring species [[9], [10], [11]]. Allelochemicals such as phenolics, flavonoids, alkaloids, terpenoids, anthraquinones, and organic acids may be released through roots, leaf litter, or foliar leachates [12,13]. These compounds can disrupt physiological processes including cell division, membrane stability, enzymatic activity, and photosynthesis, thereby influencing seed germination, root elongation, and early seedling establishment [[14], [15], [16]].

Allelopathic interference is widespread in natural and agricultural ecosystems. Aqueous extracts of *Xanthium strumarium*, for example, variably suppress germination across multiple crop species depending on concentration and plant part used for extract preparation [17]. Likewise, sorghum, black mustard (*Brassica nigra*), sunflower, and sesame release water-soluble compounds that delay germination and impair early seedling growth in both crop and wild species [[18], [19], [20], [21], [22]]. The magnitude of allelopathic effects depends on species identity, extract concentration, the plant part used, and environmental factors such as soil microbial communities and nutrient availability [[23], [24], [25]].

Beyond their ecological functions, many allelochemicals also participate in plant defense and stress responses [12,26]. Among these, anthraquinones (including emodin and aloe-emodin) occur in high concentrations in the roots and rhizomes of the *Rheum* genus and exhibit strong phytotoxic, antimicrobial, and oxidative stress-inducing activities [[27], [28], [29]]. These compounds may act as chemical barriers against competitors and pathogens, contributing to the ecological dominance of allelopathic species. *Rheum ribes* L. (wild rhubarb), a perennial herb native to mountainous and steppe rangelands of Iran, is particularly rich in anthraquinones and polyphenols such as emodin, rhein, and catechin [[30], [31], [32]], raising the possibility that its dominance may in part result from allelopathic interactions.

Despite its ecological, medicinal, and cultural significance, the allelopathic potential of *R. ribes* remains underexplored. Field observations indicate that habitats dominated by this species often exhibit reduced plant diversity, with especially sparse populations of *Artemisia* spp. and *Stipa* spp., suggesting possible allelopathic suppression. Given the limited experimental evidence, evaluating potential allelopathic effects of *R. ribes* is essential for developing informed rangeland restoration strategies, particularly in areas where chemical interference may hinder reseeding efforts or natural plant recovery [[33], [34], [35]].

The present study investigates the allelopathic effects of aqueous root and leaf extracts from *R. ribes* at two concentrations (50% and 100%) on seed germination timing and early seedling growth of two ecologically important rangeland species: *Stipa barbata* Desf. (Poaceae) and *Astragalus cyclophyllon* G. Beck (Fabaceae). Both species are well adapted to arid and semi-arid ecosystems and are considered promising candidates for rangeland rehabilitation in degraded landscapes. The timing of germination was specifically examined because it plays a critical role in determining seedling establishment, competitive interactions, and overall fitness of plant populations [36,37]. Early or delayed germination can influence survival rates, resource acquisition, and vulnerability to environmental stresses [[38], [39], [40]]. By evaluating germination timing, we can better understand how different species or populations respond to environmental cues and potential allelopathic interactions [41,42]. This information is important for predicting population dynamics, succession patterns, and species coexistence in natural and managed ecosystems [43,44]. By integrating germination metrics with species-specific growth responses, this research contributes to a deeper understanding of how allelopathy shapes recruitment dynamics in steppe ecosystems. It also provides foundations for future studies aimed at identifying active compounds through phytochemical profiling, examining their persistence in natural soils, and evaluating their implications for ecological restoration and sustainable weed management [[45], [46], [47]]. Specifically, this study addressed four key objectives: (1) to determine whether aqueous leaf and root extracts of *Rheum ribes* delayed or inhibited seed germination and germination timing in *Astragalus cyclophyllon* and *Stipa barbata*; (2) to evaluate the effects of extract concentration and plant part on early seedling growth variables, including radicle and epicotyl length and dry biomass; (3) to assess species-specific sensitivity in both germination dynamics and seedling growth responses; and (4) to evaluate the feasibility of establishing these species in *R. ribes*-dominated habitats as part of rangeland restoration strategies.

## 2 Materials and methods

### 2.1 Study area and plant material collection

**Fig. 1:**
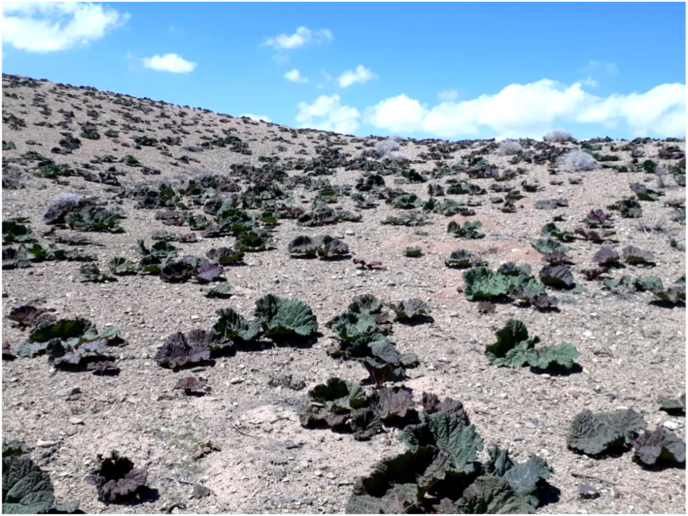
Steppe habitat of *Rheum ribes* in the Cheshme Gaz region, Kerman, Iran.

This study was conducted in the Cheshme Gaz rangelands, situated approximately 40 km northwest of Kerman city (Fig. 1). The geographical coordinates of the study area range from 56°36′32″ to 56°43′59″ E and 30°22′19″ to 30°29′27″ N. The region has an elevation range from 2011 to 2706 m, with an average elevation of approximately 2330 m and a slope of 11.11%. According to the De Martonne classification, the area experiences a dry desert climate, with an annual rainfall of 116.3 mm and a mean annual temperature of 17.3 °C. The soil in the region is primarily sandy-clayey, exhibiting high permeability and an alkaline pH [48].

In the spring of 2024, six complete specimens of *Rheum ribes* were randomly collected from the study site, encompassing both aerial (leaves) and underground (roots) parts. After transportation to the laboratory, the leaves and roots were separated and air-dried at room temperature (approximately 25 °C). Subsequently, they were ground into a fine powder using an electric grinder (IKA A 11 basic Analytical Mill).

### 2.2 Preparation of aqueous extracts

Aqueous extracts are commonly used in allelopathy studies as they mimic natural processes such as rainfall leaching and root exudation. Although water may not efficiently extract poorly soluble compounds, different studies demonstrate that water-based extracts are sufficient to reveal allelopathic effects, including reduction of germination and seedling growth [[49], [50], [51]]. This suggests that even hydrophilic allelochemicals can significantly affect target plants, supporting the use of aqueous extraction as a reliable method to assess allelopathic potential. Fresh leaves and roots were washed with distilled water, air-dried to remove surface moisture, and cut into uniform sections. Each part type was processed separately. Aqueous extracts were prepared by soaking 5 g of plant tissue in 1 L of distilled water for 24 h at room temperature (20-22 °C) in darkness to minimize degradation of light-sensitive compounds [52,53]. The mixtures were filtered through sterile muslin cloth followed by Whatman No. 1 filter paper [54,55]. The resulting solution constituted the 100% extract. A 50% concentration was prepared by diluting the stock extract with distilled water immediately before application (i.e. 5 g of dry plant tissue in 2 L of distilled water). Extracts were stored at 4 °C until use.

### 2.3 Experimental setup

Seeds of both species were surface-sterilized with 1% sodium hypochlorite for 5 min to eliminate surface pathogens and ensure aseptic germination conditions [56]. This treatment does not induce scarification. Seeds were rinsed three times with sterile distilled water and allowed to air-dry briefly. Sterilization procedures were conducted under low-light conditions to reduce oxidative stress.

A fully factorial experiment was designed with three factors: plant species (*Stipa barbata*, *Astragalus cyclophyllon*), plant part (leaf, root), and concentration (0%, 50%, 100%). The experiment followed a completely randomized design with four replications per treatment, totaling 48 Petri dishes. Each dish contained 25 seeds placed on double-layered sterile Whatman filter paper in 9-cm Petri plates. Five milliliters of the designated extract concentration were added to each dish. Control dishes received distilled water. All dishes were sealed with Parafilm to prevent moisture loss.

Petri dishes were incubated in a controlled-environment growth chamber (Model X615TC; Fater Electronics, Iran) at 20 °C with a 12 h light/12 h dark photoperiod. Dishes were rotated daily to minimize positional effects within the chamber. When necessary, very small and identical volumes of sterilized distilled water were added across all treatments to prevent desiccation.

The counting of germinated seeds began on day 1 and continued daily until day 35. Radicle and epicotyl lengths were measured using a calibrated digital caliper (IP67 Digital Caliper; Insize Co., China). Seedlings were oven-dried at 70 °C for 48 h and individually weighed using an analytical balance (Kern IoT-Line Precision Balance, 0.001 g readability; Kern and Sohn GmbH, Germany) to determine dry biomass.

The following indices were calculated: (1) T_10_, T_50_, T_90_: time to reach 10%, 50%, and 90% germination, respectively [57]; (2) Mean Germination Time (MGT): calculated using standard formulas [58]; [59] and (3) Germination Rate Index (GRI): calculated as the cumulative daily germination percentage, following Maguire [60].

### 2.4 Statistical analyses

Germination percentage, radicle and epicotyl length, and radicle and epicotyl biomass were analyzed using factorial analysis of variance (ANOVA) within the General Linear Model (GLM) framework in SPSS version 24 (IBM Corp., Armonk, NY, USA). The models included plant species, plant part (leaf vs. root), and extract concentration as fixed factors, as well as their interactions. Assumptions of normality and homogeneity of variances were checked prior to analysis.

When ANOVA results indicated significant main effects or interactions (p < 0.05), post hoc comparisons were conducted to explore differences among treatment levels. Tukey's HSD test was applied for a limited number of planned comparisons following significant ANOVA results, in order to maintain statistical power. Post hoc tests were not conducted for factors with non-significant ANOVA results.

To facilitate comparison of treatment effects relative to the control, percent change was calculated using the following formula:

Equation 1 $$\text{Percent}\;\text{change}=\frac{\text{Treatment}\;\text{value}-\text{Control}\;\text{value}}{\text{Control}\;\text{value}}\times 100$$

Percent change plots were generated using Microsoft Excel 2021.

Germination timing variables, including time to 10%, 50%, and 90% germination (T_10_, T_50_, T_90_), mean germination time (MGT), and germination rate index (GRI), were analyzed in the R statistical environment (version 4.3.1; R Core Team, 2023). Data manipulation and visualization were performed using the tidyverse suite of packages (Wickham et al., 2019). These variables were analyzed using multi-factor ANOVA to test for the effects of species, plant part, extract concentration, and their interactions. When significant effects were detected, Tukey's HSD test was applied for post hoc comparisons.

Because germination is a time-to-event process and some seeds remained ungerminated by the end of the observation period (right-censored data), germination dynamics were additionally analyzed using survival analysis methods in R. Kaplan-Meier survival curves were constructed to visualize cumulative germination over time for each treatment. Differences among curves were assessed using Cox proportional hazards models, which were used to evaluate the effects of species, plant part, extract concentration, and their interactions on the hazard of germination. Survival analyses were implemented using the survival package [61], and graphical outputs were generated using survminer [62]. All statistical tests were evaluated at a significance level of α = 0.05.

## 3 Results

### 3.1 Germination rate, radicle and epicotyl lengths and biomass

A significant main effect of plant part was detected (p = 0.002; Table 1), with root extracts (M = 22.83%) reducing germination more strongly than leaf extracts (M = 15.33%). Although the main effect of concentration was not significant (p = 0.575), a significant concentration × species interaction (p = 0.013) indicated differential species-level responses.

**Table 1:** Summary of three-way ANOVA (Species (Sp) × Part (Par) × Concentration (Conc)) for all variables.

Variable	Sp (F, p)	Par (F, p)	Conc (F, p)	Sp × Par (F, p)	Sp × Conc (F, p)	Par × Conc (F, p)	Sp × Par × Conc (F, p)
Germination Rate (G)	2.095, 0.156	11.750, 0.002	0.563, 0.575	1.306, 0.261	4.880, 0.013	0.679, 0.514	2.629, 0.086
Epicotyl Length (EL)	61.579, <0.001	0.233, 0.632	2.732, 0.079	0.503, 0.483	2.048, 0.144	0.070, 0.933	0.205, 0.816
Epicotyl Weight (EW)	13.330, 0.001	0.035, 0.852	4.134, 0.024	1.021, 0.319	0.406, 0.670	0.572, 0.569	1.252, 0.298
Radicle Length (RL)	1.116, 0.298	3.497, 0.070	3.555, 0.039	3.083, 0.088	9.637, <0.001	1.985, 0.152	0.134, 0.875
Radicle Weight (RW)	0.286, 0.596	0.631, 0.432	7.589, 0.002	0.116, 0.735	0.732, 0.488	2.514, 0.095	3.966, 0.028

**Fig. 2:**
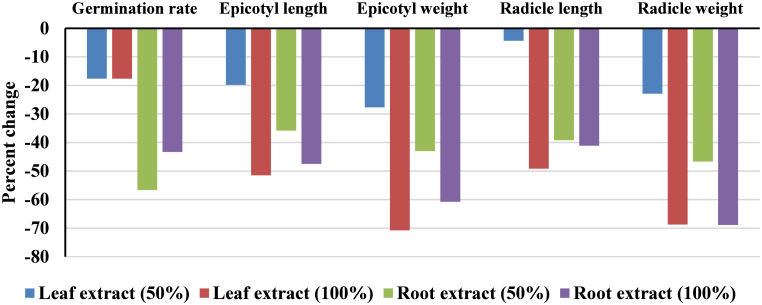
The percentage of changes in the germination rate, epicotyl length and weight, and radicle length and weight of the *Stipa barbata* with 50 and 100% *Rheum ribes* leaf and root extracts compared to the control.

In *Stipa barbata*, germination was reduced across all treatments. Relative to the control, leaf extracts reduced germination by 17.65% at both 50% and 100% concentrations (Table 2; Fig. 2). Root extracts exerted even stronger effects, causing up to a 56.67% reduction at 100% concentration (Fig. 2).

**Table 2:** Effects of *Rheum ribes* leaf and root aqueous extracts at 0% (control), 50%, and 100% concentrations on germination rate and seedling growth parameters of *Stipa barbata*. Values are expressed as mean ± standard error (SE). Different letters within rows indicate statistically significant differences at *p* < 0.05 according to post hoc tests.

Variable	Leaf extract			p	Root extract			p
	0%	50%	100%		0%	50%	100%	
Germination rate	3.42 ± 17.00	4.76 ± 14.00	3.46 ± 14.00	0.83	1.16^a^±30.00	^b^ 5.26 ± 13.00	^bc^ 3.79 ± 17.00	0.03
Epicotyl length	14.72 ± 41.00	11.46 ± 32.88	7.64 ± 19.88	0.46	5.58 ± 50.25	12.88 ± 32.25	5.95 ± 26.38	0.19
Epicotyl weight	0.0014^a^±0.0065	0.0016 ^ab^ ± 0.0047	0.0007 ^b^ ± 0.0019	0.09	0.0018 ± 0.0079	0.0018 ± 0.0045	0.0007 ± 0.0031	0.13
Radicle length	6.46 ± 22.88	7.57 ± 21.88	3.83 ± 11.63	0.39	3.38 ± 26.13	6.65 ± 15.88	2.90 ± 15.38	0.23
Radicle weight	0.0012 ± 0.0048	0.0013 ± 0.0037	0.0007 ± 0.0015	0.14	0.0002^a^ ±0.0045	^ab^ 0.0011 ± 0.0024	^b^ 0.0004 ± 0.0014	0.03

**Fig. 3:**
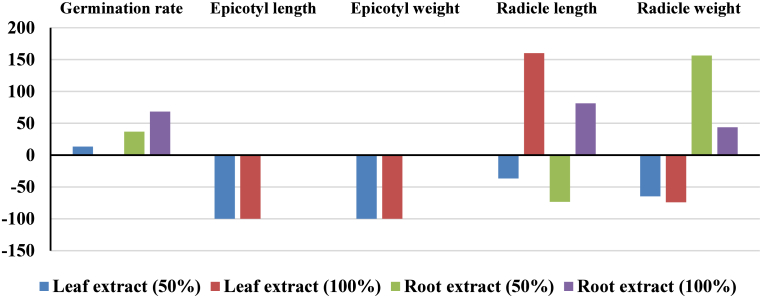
The percentage of changes in the germination rate, epicotyl length and weight, and radicle length and weight of the *Astragalus cyclophyllon* with 50 and 100% *Rheum ribes* leaf and root extracts compared to the control.

*Astragalus cyclophyllon* displayed a nonlinear response. Germination increased by 13.33% under 50% leaf extract but was not changed (0%) under 100% leaf extract (Fig. 3). Root extracts produced the opposite trend i.e. germination peaked at 32% under the 100% concentration, a 68.42% increase relative to control, suggesting a hormetic response (Table 3; Fig. 3).

**Table 3:** Effects of *Rheum ribes* leaf and root aqueous extracts at 0% (control), 50%, and 100% concentrations on germination rate and seedling growth parameters of *Astragalus cyclophyllon*. Values are expressed as mean ± standard error (SE). Different letters within rows indicate statistically significant differences at *p* < 0.05 according to post hoc tests.

Variable	Leaf extract			p	Root extract			p
	0%	50%	100%		00%	50%	100%	
Germination rate	3.42 ± 15.00	1.91 ± 17.00	5.97 ± 15.00	0.92	3.79^a^±19.00	3.83^ab^ ± 26.00	1.63^b^ ± 32.00	0.05
Epicotyl length	2.37 ± 3.13	0.00 ± 0.00	0.00 ± 0.00	0.23	0.00 ± 0.00	0.25 ± 0.25	0.00 ± 0.00	0.41
Epicotyl weight	0.0049 ± 0.0049	0.00 ± 0.00	0.00 ± 0.00	0.41	0.00 ± 0.00	0.0013 ± 0.0013	0.00 ± 0.00	0.41
Radicle length	1.51^a^±7.50	0.72^ab^ ± 4.75	5.91^b^ ± 19.50	0.03	5.59^ab^ ± 20.63	2.09^a^±5.50	7.46^b^ ± 37.38	0.01
Radicle weight	0.001^a^ ±0.0054	0.0002^b^ ± 0.0019	0.0004^bc^±0.0014	0.00	0.0006 ± 0.0016	0.0016 ± 0.0041	0.0006 ± 0.0023	0.29

Species had a highly significant effect on epicotyl length (p < 0.001; Table 1), with *S. barbata* producing substantially longer epicotyls (M = 33.77 mm) than *A. cyclophyllon* (M = 0.56 mm). Concentration showed a marginal effect (p = 0.079), and post hoc Tukey's HSD tests indicated that 100% extract significantly reduced epicotyl length relative to the control (p = 0.026). The concentration × species interaction approached significance (p = 0.144), suggesting greater sensitivity in *A. cyclophyllon*.

In *S. barbata*, epicotyl length declined by 19.80% and 51.51% under 50% and 100% leaf extracts, respectively (Fig. 2), and by 35.82% and 47.50% under root extracts (Fig. 2). In contrast, *A. cyclophyllon* exhibited complete epicotyl inhibition (−100%) in response to both concentrations of leaf extract (Fig. 3).

Epicotyl biomass (EW) was significantly influenced by concentration (p = 0.024) and species (p = 0.001) (Table 1). *Stipa barbata* accumulated more biomass (M = 0.00476 g) than *A. cyclophyllon* (M = 0.00103 g). Although the three-way interaction was not significant (p = 0.298), it showed strong patterns consistent with differential species tolerance.

In *S. barbata*, epicotyl weight decreased by 27.69% and 70.77% under 50% and 100% leaf extracts, respectively, and by 43.04% and 60.76% under root extracts (Fig. 2). In *A. cyclophyllon*, epicotyl weight was reduced by 100% under all leaf extract treatments and remained near zero under most root extract conditions (Fig. 3).

Radicle length was significantly affected by concentration (p = 0.039), and the concentration × species interaction was highly significant (p < 0.001; Table 1). *Stipa barbata* maintained relatively stable radicle lengths across treatments, whereas *A. cyclophyllon* displayed a combination of inhibition and stimulation.

In *S. barbata*, radicle length declined by 4.37% and 49.17% under 50% and 100% leaf extracts, respectively, and by 39.23% and 41.14% under root extracts (Fig. 2). In contrast, *A. cyclophyllon* radicle length decreased by 36.67% under 50% leaf extract but unexpectedly increased by 160% under 100% leaf extract (Fig. 3), indicating potential hormetic stimulation. Root extracts caused 73.34% reduction in radicle length under 50% leaf extract but 81.19% increase under 100% root extract in *A. cyclophyllon* (Fig. 3).

Radicle biomass (RW) was significantly influenced by concentration (p = 0.002), and a significant concentration × part × species interaction (p = 0.028) was detected (Table 1). Post hoc tests confirmed heavy reductions at 100% concentration relative to both control (p < 0.001) and 50% (p = 0.033).

In *S. barbata*, radicle weight declined by 22.92% and 68.75% under 50% and 100% leaf extracts, with up to 68.89% reduction under 100% root extract (Fig. 2). In *A. cyclophyllon*, radicle biomass declined by 64.81% and 74.07% under 50% and 100% leaf extracts, with up to 156.25% increase under 50% root extract (Fig. 3).

### 3.2 Germination times (T_10_, T_50_, T_90_) and cumulative germination trend

**Fig. 4:**
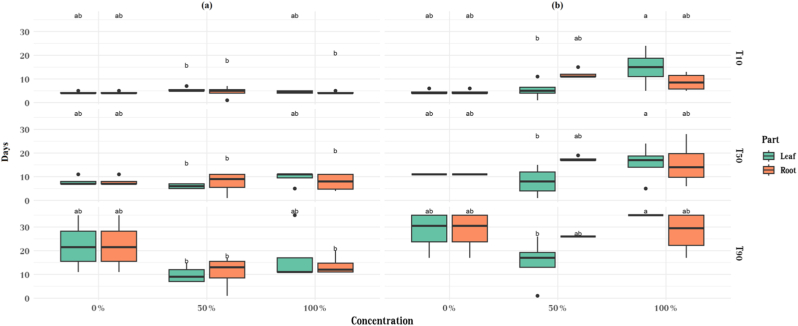
Comparison of germination times (T10, T50, and T90) in *Astragalus cyclophyllon* (a) and *Stipa barbata* (b), influenced by different plant parts (leaf and root) and extract concentrations.

The effects of species, plant part, and concentration on the measured response were evaluated using three-way ANOVA (Table 4) and Tukey HSD post-hoc tests (Fig. 4). For T10, ANOVA revealed significant effects of species (F = 18.95, p < 0.001) and concentration (F = 6.23, p = 0.0048), as well as significant interactions including Species × Concentration (F = 5.58, p = 0.0078), Part × Concentration (F = 3.80, p = 0.0319), and the three-way interaction Species × Part × Concentration (F = 4.85, p = 0.0137). Tukey HSD comparisons showed that *S*. *barbata* generally produced higher responses than *A*. *cyclophyllon*, with specific contrasts between roots and leaves of different species reaching significance. For T50, only species had a significant effect (F = 16.62, p < 0.001), while concentration, plant part, and their interactions were not significant. Tukey HSD tests confirmed the significant difference between *S*. *barbata* and *A*. *cyclophyllon*, although fewer pairwise differences were observed compared with T10. For T90, species showed a significant main effect (F = 7.13, p = 0.011), whereas plant part, extract concentration, and their interactions were not significant (p > 0.05).

**Table 4:** ANOVA results for T10, T50, and T90 showing the effects of species, concentration, and their interactions on the measured variables.

Factor	Df	T10		T50		T90	
		F	p	F	p	F	p
Species	1	18.949	0.000	16.616	0.000	7.132	0.011
Part	1	0.020	0.886	1.314	0.259	1.018	0.319
Concentration	2	6.226	0.004	1.634	0.209	2.106	0.136
Species:Part	1	0.112	0.739	1.464	0.234	1.050	0.312
Species:Concentration	2	5.576	0.007	0.843	0.438	1.836	0.175
Part:Concentration	2	3.796	0.031	2.385	0.106	1.342	0.272
Species:Part:Concentration	2	4.849	0.013	0.880	0.423	0.791	0.460

**Fig. 5:**
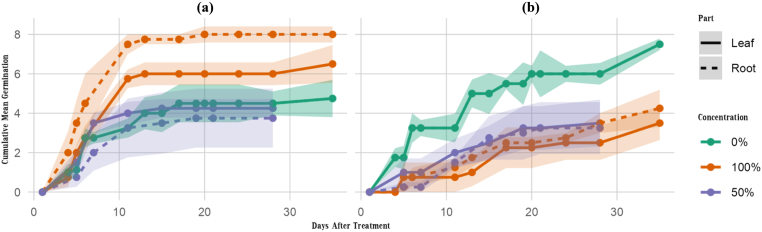
Cumulative mean germination over time for different concentrations and plant parts in *Astragalus cyclophyllon* (a) and *Stipa barbata* (b). Values represent the mean cumulative germination across replicates, and shaded ribbons indicate ± standard error (SE). Germination was recorded daily following treatment. Lines are constrained to be non-decreasing to reflect cumulative germination dynamics.

Cumulative germination curves for each species, plant part, and concentration are shown in Fig. 5. These curves illustrate temporal patterns of germination and highlight clear treatment-level differences. Higher extract concentrations (and particularly root-derived extracts) generally slowed or suppressed cumulative germination in both species, although *A. cyclophyllon* showed variable trends consistent with hormetic responses.

### 3.3 Mean Germination Time (MGT) and Germination Rate Index (GRI)

**Fig. 6:**
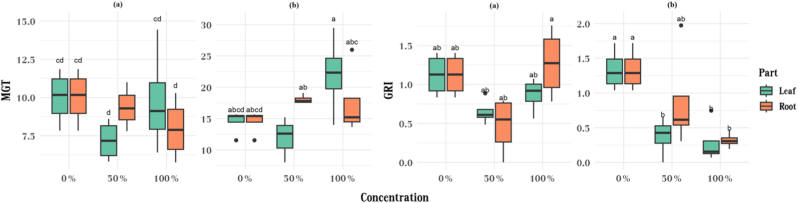
Comparison of Mean Germination Time (MGT) and Germination Rate Index (GRI) in *Astragalus cyclophyllon* (a) and *Stipa barbata* (b) affected by different plant parts (leaf and root) and extract concentrations.

Both MGT and GRI varied according to species, plant part, and extract concentration (Table 5). MGT generally increased with extract concentration, especially in *S. barbata* leaves, where 100% extracts raised MGT from 12-19 days to 21-29 days. In *A. cyclophyllon*, increases were moderate but followed the same trend (Fig. 6).

**Table 5:** ANOVA results for Germination Rate Index (GRI) and Mean Germination Time (MGT) showing the effects of species, concentration, and their interactions on the measured traits.

Factor	Df	GRI		MGT	
		F	p	F	p
Species	1	2.588	0.116	64.827	2.19E-09
Part	1	1.571	0.218	0.018	0.895
Concentration	2	15.10	1.73E-05	2.750	0.078
Species:Part	1	0.257	0.615	0.016	0.899
Species:Concentration	2	8.982	0.000	4.053	0.026
Part:Concentration	2	0.419	0.661	4.853	0.014
Species:Part:Concentration	2	2.228	0.122	1.006	0.376

GRI responses were species-specific. In *A. cyclophyllon*, the highest GRI values occurred under 100% root extract, indicating accelerated germination. In contrast, *S. barbata* showed near-zero GRI at 100% extract concentration, indicating suppressed germination speed (Fig. 6).

### 3.4 Kaplan-Meier survival model analysis

**Fig. 7:**
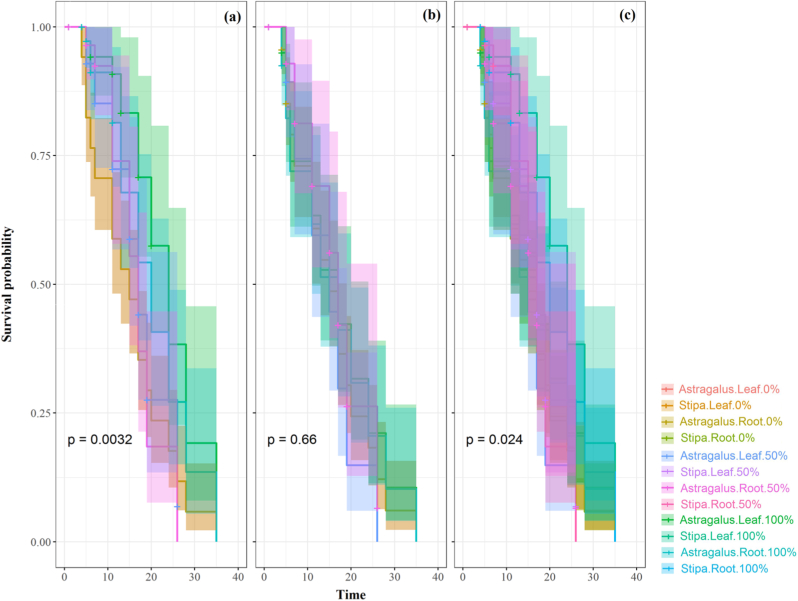
Kaplan-Meier survival curves and Cox proportional hazards analysis. (a) Survival curves for *Astragalus cyclophyllon* across different parts and concentrations. (b) Survival curves for *Stipa barbata* across different parts and concentrations. (c) Combined survival curves for all groups (Species × Part × Concentration). Shaded areas represent 95% confidence intervals; p-values are from log-rank tests.

Kaplan-Meier survival curves (Fig. 7) showed significant differences among groups (p = 0.0032). *S. barbata* exhibited higher survival probabilities (i.e., failure to germinate) under high-concentration root extracts, whereas *A. cyclophyllon* showed less pronounced effects. Pairwise log-rank tests with Benjamini-Hochberg correction identified multiple significant contrasts (Appendix A), especially involving high-concentration leaf treatments. These results confirm that species, plant part, and concentration shape germination survival dynamics. The differences observed, particularly at higher extract concentrations, indicate a substantial impact on seed survival and germination rates (Fig. 7).

### 3.5 Cox proportional hazards model

The Cox model indicated no significant effect of species or plant part on germination hazard, whereas 100% extract concentration significantly reduced germination hazard (HR = 0.698, p = 0.0008), confirming delayed germination at high concentrations. The 50% concentration had no significant effect (Table 6).

**Table 6:** Cox regression model results for species, part, and concentration effects on germination hazard, presenting the regression coefficients (β), standard errors (SE), hazard ratios (HR) with their 95% confidence intervals, z-statistic values, and p-values.

Variable	Coefficient	95% CI Lower	95% CI Upper	Std. Error	z value	p-value	Hazard Ratio (HR)
**SpeciesStipa**	−0.111	−0.289	0.067	0.091	−1.225	0.221	0.895
**PartRoot**	0.017	−0.161	0.195	0.091	0.188	0.851	1.017
**Concentration50%**	0.036	−0.209	0.281	0.125	0.288	0.773	1.037
**Concentration100%**	−0.359	−0.569	−0.149	0.107	−3.345	<0.001	0.698

Univariate Cox models (Table 7) confirmed these findings, with concentration emerging as the strongest predictor of germination hazard.

**Table 7:** Results of univariate Cox regression models for the effects of species, part, and concentration on survival time.

Variable	Coefficient	95% CI Lower	95% CI Upper	Std. Error	z value	p-value	Hazard Ratio (HR)
**SpeciesStipa**	−0.1124	0.748	1.068	0.0909	−1.237	0.216	0.8937
**PartRoot**	0.0181	0.852	1.216	0.0907	0.199	0.842	1.018
**Concentration50%**	0.0358	0.811	1.324	0.1249	0.287	0.774	1.036
**Concentration100%**	−0.3595	0.566	0.862	0.1073	−3.349	0.0008	0.698

A forest plot (Appendix B) visualizes hazard ratios across predictors, highlighting the dominant inhibitory effect of 100% extract concentration.

## 4 Discussion

### 4.1 Species-, organ-, and concentration-dependent allelopathic effects on germination rate and seedling growth parameters

This study examined the allelopathic effects of aqueous leaf and root extracts of *Rheum ribes* at two concentrations (50% and 100%) on germination timing and early seedling development of two ecologically important steppe species, *Stipa barbata* and *Astragalus cyclophyllon*. The results clearly demonstrate that allelopathic effects imposed by *R. ribes* are strongly species-specific, plant part-dependent, and concentration-dependent, with complex interactive patterns emerging from these factors. While *S. barbata* exhibited a predominantly inhibitory and stress-sensitive response, *A. cyclophyllon* displayed a more nuanced pattern, including both inhibition and hormetic stimulation under specific treatments. Together, these findings provide new insights into the chemical ecology of *R*. *ribes*-dominated rangelands and have implications for rangeland restoration planning and species selection.

Across all measured variables, *S. barbata* showed a consistent and largely inhibitory response to both leaf and root extracts of *R. ribes*. Although not all effects reached statistical significance, germination rate, epicotyl length, epicotyl biomass, radicle length, and radicle biomass were all reduced to varying degrees. Notably, root extracts at higher concentrations caused the most severe inhibition, with germination decreasing by more than 56% and radicle biomass by nearly 69%. These findings align with previous reports demonstrating the sensitivity of Poaceae species to phenolic- and terpenoid-rich allelochemicals [63,64].

The significant main effects of extract concentration and the concentration × species interactions observed for epicotyl and radicle traits indicate that *S. barbata* is particularly susceptible to chemical interference under high extract exposure. The near-complete suppression of epicotyl elongation and biomass suggests impairment of shoot meristem activity, potentially associated with a mixture of secondary metabolites reported for *R. ribes*, including flavonoids, tannins, and other phenolic compounds, while less water-soluble constituents such as anthraquinones may contribute indirectly or at low effective concentrations within complex aqueous extracts. These metabolites are known to interfere with hormonal signaling pathways and cellular respiration [65,66], which may explain the impaired seedling development observed even among germinated seeds.

In contrast to *S. barbata*, *A. cyclophyllon* displayed a more complex and variable response to *R. ribes* extracts. Germination and radicle length were significantly stimulated by root extracts, with increases of up to 68.42% and 160%, respectively. This pattern is consistent with hormesis, a phenomenon whereby low or moderate levels of stress-inducing compounds stimulate physiological performance ([67]; Takao et al., 2011).

The relative tolerance of *A. cyclophyllon* may be related to its robust antioxidant defense systems and potential synergistic interactions with rhizobia, which can buffer oxidative stress and enhance nutrient acquisition [68,69]. However, this tolerance was clearly tissue-specific. While radicle growth was promoted under certain treatments, epicotyl development was entirely inhibited across all extract concentrations, particularly at 100%. This pattern was reflected in the significant three-way interaction (concentration × plant part × species) observed for radicle biomass, illustrating that even within a single species, different tissues may respond divergently to allelochemical exposure.

Such divergence suggests that although *A. cyclophyllon* may successfully germinate and establish root systems under chemical stress, aboveground development could remain constrained, potentially limiting long-term establishment and competitive performance under field conditions.

### 4.2 Species-, organ-, and concentration-dependent allelopathic effects on germination timing, germination indices, and survival analysis

The allelopathic responses observed in this study further indicate that germination timing is strongly influenced by species identity and, to a lesser extent, by extract concentration and plant part. The highly significant species effect detected for T_10_, T_50_, and T_90_ suggests inherent differences in germination biology between *S. barbata* and *A. cyclophyllon*, rather than differential sensitivity to allelochemical stress. Species-specific differences in germination responses have been widely reported in the literature, including in allelopathic studies. However, such differences often reflect intrinsic biological variation in germination traits rather than solely differential sensitivity to allelochemical stress [70,71].

For T_10_, extract concentration exerted a significant effect, indicating a clear dose-dependent response during early germination. Early germination stages are particularly sensitive to allelochemicals because radicle protrusion coincides with high metabolic activity and rapid water uptake [72,73]. The significant species × plant part × concentration interaction further suggests that allelopathic expression depends on the combined contribution of plant identity, organ-specific metabolite allocation, and extract strength, consistent with reports that roots and leaves often differ markedly in secondary metabolite composition [74,75].

Tukey comparisons for T_10_ showed that *S. barbata*, particularly under 100% extract concentration, experienced the strongest inhibitory effects, whereas *A. cyclophyllon* exhibited weaker or non-significant responses.

In contrast, T_50_ displayed a persistent species effect but weaker concentration dependence. This reduced sensitivity at later germination stages may reflect degradation or dilution of allelochemicals over time, as well as increasing physiological tolerance as seedlings transition from early to mid-developmental stages [76,77]. Again, stronger inhibition by *S. barbata*, particularly in root extract treatments, supports the view that allelopathic roots may exert substantial influence on belowground competitive interactions.

Consistent with these patterns, aqueous extracts of *R. ribes* exerted measurable effects on germination performance, with responses differing markedly across species, plant parts, and extract concentrations. The strong main effect of extract concentration on the Germination Rate Index (GRI) confirms that allelopathic inhibition is dose-dependent, in line with the established principle that allelochemical activity increases with extract strength or concentration of released metabolites [[78], [79], [80]].

Although the main effects of species and plant part on GRI were not significant, the highly significant species × concentration interaction highlights contrasting sensitivities between the two species. Such differences may reflect variation in seed coat permeability, detoxification capacity, or intrinsic germination physiology [81,82]. Mean Germination Time (MGT) provided complementary insight into temporal germination dynamics, revealing intrinsic species-level differences in germination speed and context-dependent allelopathic delays shaped by extract concentration and plant part.

Survival analyses further supported these interpretations. Kaplan-Meier curves and Cox proportional hazards models showed that *S. barbata* seeds had a substantially higher probability of remaining ungerminated under high-concentration root extracts, whereas *A. cyclophyllon* exhibited no comparable survival penalty. The significant three-way interaction (species × plant part × concentration) observed for radicle biomass reinforces the conclusion that *R. ribes* imposes differential allelopathic pressure across species and tissues.

### 4.3 Mechanistic considerations and ecological relevance

The inhibitory effects observed in *S. barbata* are consistent with the phytotoxic potential of secondary metabolites reported in *R. ribes*. Phytochemical studies have identified a broad spectrum of compounds in the roots and leaves of this species, including phenolic acids, flavonoids, tannins, and anthraquinones such as emodin [83,84]. However, because this study relied exclusively on aqueous extracts, the observed effects should be interpreted as arising from the combined action of water-soluble and partially soluble constituents rather than from individual compounds. While anthraquinones are generally considered poorly soluble in water, their apparent bioavailability in natural extracts may be enhanced through interactions with co-occurring metabolites such as organic acids, phenolics, polysaccharides, or saponin-like compounds acting as natural solubilizers. Alternatively, hydrophilic compounds alone may account for much of the observed inhibition, with less soluble metabolites contributing indirectly or at low effective concentrations.

The stimulatory responses observed in *A. cyclophyllon* radicles are consistent with hormesis, documented in legumes and other stress-adapted taxa [85]. However, the complete inhibition of epicotyl growth despite radicle stimulation indicates pronounced tissue-specific sensitivity, potentially reflecting differential detoxification capacity or hormonal regulation between root and shoot tissues. The hypocotyl-epicotyl transition zone may be particularly vulnerable to allelochemical interference [86].

### 4.4 Implications for rangeland ecology, management, and restoration

Ecologically, the strong suppression of *S. barbata*, a key perennial grass, suggests that *R. ribes* may substantially hinder grass recruitment within its dominant patches. Reduced grass establishment could compromise soil stabilization and forage production, given the functional importance of Poaceae in steppe ecosystems [3]. In contrast, the relative tolerance of *A. cyclophyllon* suggests that legumes may be more capable of establishing in *R. ribes*-dominated habitats and may contribute to ecosystem recovery through nitrogen enrichment and microhabitat modification.

From a management perspective, these findings highlight both the potential and the risks of leveraging allelopathic interactions in vegetation management or bioherbicide development. Because allelochemicals can suppress some species while stimulating others, their application requires careful consideration to avoid unintended ecological consequences.

## 5 Conclusion

This study demonstrated that aqueous leaf and root extracts of *Rheum ribes* exerted clear allelopathic effects on seed germination dynamics and early seedling growth of two native rangeland species. The magnitude and direction of these effects depended strongly on species identity, plant part, and extract concentration. Overall, *Stipa barbata* showed high sensitivity, expressed as delayed germination and reduced seedling growth, particularly under root-derived extracts, whereas *Astragalus cyclophyllon* exhibited more variable responses, including both inhibitory and stimulatory effects on radicle development. Importantly, these findings emphasize that allelopathic interference by *R. ribes* should be considered in rangeland restoration and revegetation programs, especially when selecting species for reseeding in *R. ribes*-dominated habitats. While the present results reflect the integrated effects of complex aqueous extracts rather than individual compounds, they provide a foundation for future studies combining phytochemical characterization with field-based experiments to better assess the ecological relevance of allelopathy under natural conditions.

## AI-use disclosure statement

Portions of this manuscript (text editing, grammatical revision, and formatting) were assisted by generative AI (ChatGPT, OpenAI). All scientific interpretation, data analysis, and conclusions were produced by the authors.

## Funding

This work was supported by the 10.13039/100019273University of Jiroft and was conducted as part of a MSc thesis.

## CRediT authorship contribution statement

**Saeed Sheikh Ali Babaei Mahani:** Conceptualization, Data curation, Investigation, Methodology, Writing – original draft, Writing – review & editing. **Mohsen Sharafatmandrad:** Conceptualization, Formal analysis, Methodology, Project administration, Software, Supervision, Visualization, Writing – original draft, Writing – review & editing. **Esfandiar Jahantab:** Software, Writing – original draft, Writing – review & editing.

## Declaration of competing interest

The authors declare that they have no known competing financial interests or personal relationships that could have appeared to influence the work reported in this paper.

## Figures and Tables

**Table:** Results of pairwise comparisons between groups using the Log-rank test with Benjamini-Hochberg correction (BH) to assess significant differences in survival curves. Groups with p-values less than 0.05 indicate significant differences in survival rates.

Group1	Group2	p	Group1	Group2	p
Astragalus.Leaf.50%	Stipa.Leaf.100%	0.01	Stipa.Root.50%	Astragalus.Leaf.50%	0.75
Stipa.Leaf.100%	Astragalus.Leaf.0%	0.01	Astragalus.Leaf.50%	Stipa.Leaf.0%	0.75
Stipa.Leaf.100%	Stipa.Leaf.0%	0.01	Stipa.Leaf.50%	Stipa.Leaf.0%	0.75
Stipa.Leaf.100%	Astragalus.Root.0%	0.01	Astragalus.Leaf.50%	Stipa.Root.0%	0.75
Stipa.Leaf.100%	Stipa.Root.0%	0.01	Stipa.Leaf.50%	Stipa.Root.0%	0.75
Stipa.Root.50%	Stipa.Leaf.100%	0.01	Stipa.Root.50%	Astragalus.Leaf.100%	0.75
Astragalus.Leaf.50%	Stipa.Root.100%	0.04	Astragalus.Root.100%	Astragalus.Leaf.0%	0.76
Astragalus.Root.50%	Stipa.Leaf.100%	0.06	Astragalus.Root.100%	Astragalus.Root.0%	0.76
Stipa.Leaf.50%	Stipa.Leaf.100%	0.08	Stipa.Root.50%	Astragalus.Root.100%	0.77
Astragalus.Root.100%	Stipa.Leaf.100%	0.10	Stipa.Leaf.50%	Astragalus.Leaf.0%	0.80
Stipa.Root.100%	Stipa.Leaf.0%	0.12	Astragalus.Root.50%	Stipa.Leaf.0%	0.80
Stipa.Root.100%	Stipa.Root.0%	0.12	Stipa.Leaf.50%	Astragalus.Root.0%	0.80
Stipa.Leaf.100%	Astragalus.Leaf.100%	0.12	Astragalus.Root.50%	Stipa.Root.0%	0.80
Stipa.Root.100%	Astragalus.Leaf.0%	0.15	Stipa.Root.50%	Stipa.Leaf.50%	0.80
Stipa.Root.100%	Astragalus.Root.0%	0.15	Astragalus.Root.50%	Astragalus.Leaf.0%	0.91
Stipa.Root.50%	Stipa.Root.100%	0.16	Astragalus.Root.50%	Astragalus.Root.0%	0.91
Astragalus.Leaf.50%	Astragalus.Leaf.100%	0.42	Astragalus.Root.50%	Astragalus.Leaf.100%	0.92
Astragalus.Root.50%	Stipa.Root.100%	0.42	Stipa.Root.50%	Astragalus.Root.50%	0.92
Astragalus.Leaf.50%	Astragalus.Root.100%	0.49	Stipa.Leaf.0%	Astragalus.Leaf.0%	0.97
Stipa.Leaf.50%	Stipa.Root.100%	0.49	Stipa.Root.0%	Astragalus.Leaf.0%	0.97
Stipa.Leaf.50%	Astragalus.Leaf.50%	0.49	Astragalus.Root.0%	Stipa.Leaf.0%	0.97
Stipa.Root.100%	Astragalus.Root.100%	0.59	Stipa.Root.50%	Stipa.Leaf.0%	0.97
Astragalus.Root.50%	Astragalus.Leaf.50%	0.63	Stipa.Root.0%	Astragalus.Root.0%	0.97
Stipa.Root.100%	Astragalus.Leaf.100%	0.65	Stipa.Root.50%	Stipa.Root.0%	0.97
Stipa.Root.100%	Stipa.Leaf.100%	0.65	Astragalus.Root.100%	Astragalus.Leaf.100%	0.97
Astragalus.Leaf.100%	Stipa.Leaf.0%	0.75	Stipa.Leaf.50%	Astragalus.Leaf.100%	0.97
Astragalus.Leaf.100%	Stipa.Root.0%	0.75	Astragalus.Root.50%	Astragalus.Root.100%	0.97
Astragalus.Leaf.100%	Astragalus.Leaf.0%	0.75	Astragalus.Root.50%	Stipa.Leaf.50%	0.97
Astragalus.Leaf.50%	Astragalus.Leaf.0%	0.75	Astragalus.Root.0%	Astragalus.Leaf.0%	1.00
Astragalus.Root.100%	Stipa.Leaf.0%	0.75	Stipa.Root.50%	Astragalus.Leaf.0%	1.00
Astragalus.Leaf.100%	Astragalus.Root.0%	0.75	Stipa.Root.0%	Stipa.Leaf.0%	1.00
Astragalus.Leaf.50%	Astragalus.Root.0%	0.75	Stipa.Root.50%	Astragalus.Root.0%	1.00
Astragalus.Root.100%	Stipa.Root.0%	0.75	Stipa.Leaf.50%	Astragalus.Root.100%	1.00

**Fig:**
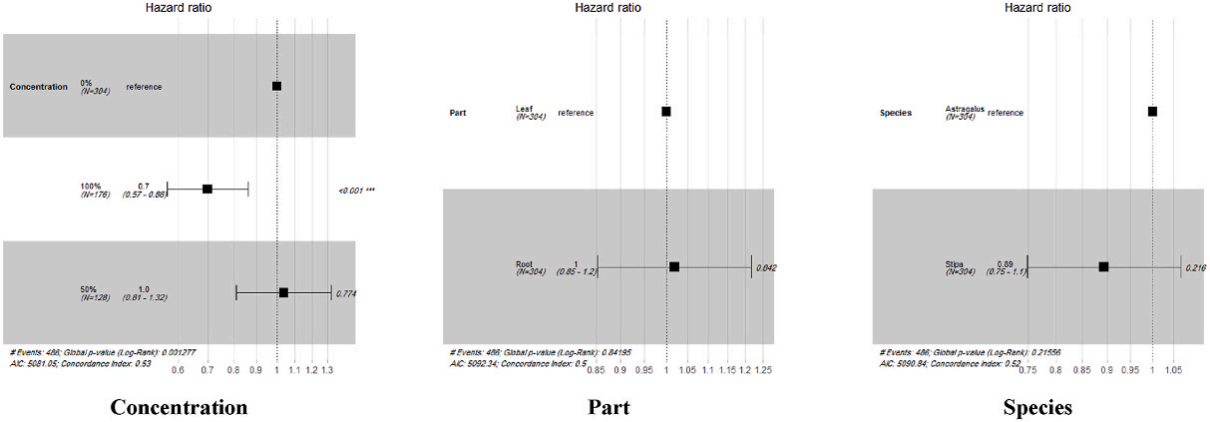
Forest plot of the Cox regression model showing the effect of Species, Part, and Concentration on sample survival. Each horizontal line indicates the 95% confidence interval of the hazard ratio (HR) for each variable level. The central point shows the HR estimate, where HR < 1 implies reduced risk, and HR > 1 indicates increased risk. The variable Concentration significantly influenced survival, with the 100% group showing a notably lower risk (HR = 0.70, p = 0.0008). Species and Part did not show statistically significant effects (p > 0.05).

## Data Availability

Data will be made available on request.

## References

[1] Mahdavi S.K., Shahraki M., Sharafatmandrad M. (2024). Consequences of participatory behavior of pastoralists on rangeland restoration. Rangel. Ecol. Manag..

[2] Khosravi Mashizi A., Sharafatmandrad M. (2021). Investigating tradeoffs between supply, use and demand of ecosystem services and their effective drivers for sustainable environmental management. J. Environ. Manag..

[3] Sala O.E., Yahdjian L., Havstad K., Aguiar M.R., Briske D.D. (2017). Rangeland Systems: Processes, Management and Challenges.

[4] Simba L.D., te Beest M., Hawkins H.J., Bowker M.A., Strydom T. (2024). Wilder rangelands as a natural climate opportunity: linking climate action to biodiversity conservation and social transformation. Ambio.

[5] Zerga B. (2015). Rangeland degradation and restoration: a global perspective. J. Agriculture. Biotechnol. Res..

[6] Wilcox B.P., Thurow T.L. (2006). Emerging issues in rangeland ecohydrology: vegetation change and the water cycle. Rangel. Ecol. Manag..

[7] Pilliod D.S., Beck J.L., Duchardt C.J., Rachlow J.L., Veblen K.E. (2022). Leveraging rangeland monitoring data for wildlife: from concept to practice. Rangelands..

[8] Lalljee B., Facknath S., Narwal S.S., Hoagland R.E., Dilday R.H., Reigosa M.J. (2000). Allelopathy in Ecological Agriculture and Forestry.

[9] Molisch H. (1937).

[10] Rice E.L. (1984).

[11] Lotina-Hennsen B., King-Díaz B., Aguilar M.I., Hernández Terrones M.G., Reigosa M.J., Pedrol N., González L. (2006). Allelopathy: a Physiological Process with Ecological Implications.

[12] Latif S., Chiapusio G., Weston L.A., Becard G. (2017).

[13] Żak A., Kosakowska A. (2016). Cyanobacterial and microalgal bioactive compounds - the role of secondary metabolites in allelopathic interactions. Oceanol. Hydrobiol. Stud..

[14] Shadab M., Bhatti N., Ain Q., Akhtar N., Parveen U., Alharby H.F., Hakeem K.R., Siddiqui M.B. (2024). Allelopathy for the sustainable management of agricultural pests: appraisal of major allelochemicals and mechanisms underlying their actions. South Afr. J. Bot..

[15] Amiri H., Mohtasham H.M. (2024). Allelopathy and its physiological mechanisms of effectiveness in plants. J. Plant. Environ. Physiol..

[16] Gniazdowska A., Krasuska U., Andrzejczak O., Soltys D., Gupta K.J., Igamberdiev A.U. (2015). Reactive Oxygen and Nitrogen Species Signaling and Communication in Plants.

[17] Mirzaee M., Saeedipour S. (2021). Allelopathic effects of *Xanthium strumarium* L. on germination and seedling growth of mung bean (*Vigna radiata* L. wilczek). Iranian J. Pulses Res..

[18] Moosavi A., Afshari R.T., Asadi A., Gharineh M.H. (2011). Allelopathic effects of aqueous extract of leaf, stem and root of sorghum (*Sorghum bicolor*) on seed germination and seedling growth of mung bean (*Vigna radiata* L.). Not. Sci. Biol..

[19] Zhao J., Yang Z., Zou J., Qu R., Li Y., Li W., Wu X. (2022). Allelopathic effects of sesame extracts on seed germination of moso bamboo and identification of potential allelochemicals. Sci. Rep..

[20] Turk M.A., Tawaha A.M. (2003). Allelopathic effect of black mustard (*Brassica nigra* L.) on germination and growth of wild oat (*Avena fatua* L.). Crop Prot..

[21] Rawat L.S., Maikhuri R.K., Bahuguna Y.M., Jha N.K., Phondani P.C. (2017). Sunflower allelopathy for weed control in agriculture systems. J. Crop Sci. Biotechnol..

[22] Bogatek R., Gniazdowska A., Zakrzewska W., Oracz K. (2006). Allelopathic effects of sunflower extracts on mustard seed germination and seedling growth. Biol. Plantarum.

[23] Al-Qthanin R., Radwan A.M., Donia A.M., Abou-Zied K.A., Balah M.A. (2024). Plant and soil characteristics affected by the allelopathic pathways of *Avena fatua* and *Lolium temulentum* weeds. Heliyon..

[24] Boutagayout A., Belmalha S., Hamdani A., Benabderrahmane A., Adiba A., Ezrari S., Nassiri L., Bouiamrine E.H. (2024). Phytochemical analysis of crop extracts and assessment of their allelopathic effect on germination and seedling growth of wild mustard (*Sinapis arvensis*) and faba bean (*Vicia faba* var. *minor*). Ecological Frontiers.

[25] Choudhary C.S., Behera B., Raza M.B., Mrunalini K., Bhoi T.K., Lal M.K., Nongmaithem D., Pradhan S., Song B., Das T.K. (2023). Mechanisms of allelopathic interactions for sustainable weed management. Rhizosphere..

[26] Akbar R., Sun J., Bo Y., Khattak W.A., Khan A.A., Jin C., Zeb U., Ullah N., Abbas A., Liu W., Wang X., Khan S.M., Du D. (2024). Understanding the influence of secondary metabolites in plant invasion strategies: a comprehensive review. Plants.

[27] Duval J., Pecher V., Poujol M., Lesellier E. (2016). Research advances for the extraction, analysis and uses of anthraquinones: a review. Ind. Crop. Prod..

[28] Izhaki I. (2002). Emodin - a secondary metabolite with multiple ecological functions in higher plants. New Phytol..

[29] Hu B., Zhang H., Meng X., Wang F., Wang P. (2014). Aloe-emodin from rhubarb (*Rheum rhabarbarum*) inhibits lipopolysaccharide-induced inflammatory responses in RAW264.7 macrophages. J. Ethnopharmacol..

[30] Kolodziejczyk-Czepas J., Liudvytska O. (2021). *Rheum rhaponticum* and *Rheum rhabarbarum*: a review of phytochemistry, biological activities and therapeutic potential. Phytochem. Rev..

[31] Chunmei D., Jiabo W., Weijun K., Cheng P., Xiaohe X. (2010). Investigation of anti-microbial activity of catechin on *Escherichia coli* growth by microcalorimetry. Environ. Toxicol. Pharmacol..

[32] Tripathi B., Bhatia R., Pandey A., Gaur J., Chawla G., Walia S., Choi E.H., Attri P. (2014). Potential antioxidant anthraquinones isolated from *Rheum emodi* showing nematicidal activity against *Meloidogyne incognita*. J. Chem..

[33] Saberi M., Shahriari A., Tarnian F., Tavili A. (2011). Influence of some chemical compounds on germination and early seedling growth of two range species under allelopathic conditions. Front. Agric. China..

[34] Sánchez-Moreiras A.M., Weiss O.A., Reigosa M.J. (2003). Allelopathic evidence in the Poaceae. Bot. Rev..

[35] Belz R.G., Duke S.O. (2014). Herbicides and plant hormesis. Pest Manag. Sci..

[36] Zacchello G., Vinyeta M., Ågren J. (2020). Strong stabilizing selection on timing of germination in a Mediterranean population of *Arabidopsis thaliana*. Am. J. Bot..

[37] Rühl A.T., Donath T.W., Otte A., Eckstein R.L. (2016). Impacts of short-term germination delay on fitness of the annual weed *Agrostemma githago* (L.). Seed Sci. Res..

[38] De Souza A.C., Donohue K., De Mattos E.A. (2022). The effect of seed-dispersal timing on seedling recruitment is modulated by environmental conditions that vary across altitude in a threatened palm. Ann. Bot..

[39] Pascarella J.B. (2024). Seeds vs. seedlings: the long-term success of seeds versus seedlings in the restoration of the federally endangered *Baptisia arachnifera* (Fabaceae) during experimental planting. Seeds..

[40] Simons A.M., Johnston M.O. (2006). Environmental and genetic sources of diversification in the timing of seed germination: implications for the evolution of bet hedging. Evolution.

[41] Maleki K., Baskin C.C., Baskin J.M., Kiani M., Alahdadi I., Soltani E. (2022). Seed germination thermal niche differs among nine populations of an annual plant: a modeling approach. Ecol. Evol..

[42] Gremer J.R., Chiono A., Suglia E., Bontrager M., Okafor L., Schmitt J. (2020). Variation in the seasonal germination niche across an elevational gradient: the role of germination cueing in current and future climates. Am. J. Bot..

[43] Postma F.M., Lundemo S., Ågren J. (2016). Seed dormancy cycling and mortality differ between two locally adapted populations of *Arabidopsis thaliana*. Ann. Bot..

[44] Gioria M., Pyšek P., Osborne B.A. (2018). Timing is everything: does early and late germination favor invasions by herbaceous alien plants?. J. Plant Ecol..

[45] Janusauskaite D. (2023). The allelopathic activity of aqueous extracts of *Helianthus annuus* L., grown in boreal conditions, on germination, development, and physiological indices of *Pisum sativum* L. Plants.

[46] Šoln K., Klemenčič M., Koce J.D. (2022). Plant cell responses to allelopathy: from oxidative stress to programmed cell death. Protoplasma..

[47] Calabrese E.J., Blain R.B. (2009). Hormesis and plant biology. Environ. Pollut..

[48] Hamzeh-Nezhadi A. (2022). Forests, Range and Watershed Management Organization (FRWMO), Kerman.

[49] Liu J., Liu X., Fu S., Wang H., Mu L. (2025). Allelopathic impact of *Erigeron canadensis* and *Erigeron annuus* on major crop species. Diversity.

[50] Shan Z., Zhou S., Shah A., Arafat Y., Rizvi S.A.H., Shao H. (2023). Plant allelopathy in response to biotic and abiotic factors. Agronomy..

[51] Wang C., Liu Z., Wang Z., Pang W., Zhang L., Wen Z., Zhao Y., Sun J., Wang Z.Y., Yang C. (2022). Effects of autotoxicity and allelopathy on seed germination and seedling growth in *Medicago truncatula*. Front. Plant Sci..

[52] Jalali M., Moosarinasab M., Saffari M. (2013). Allelopathic effects of aqueous extracts of *Xanthium strumarium* L. on germination characteristics and seedling growth of *zea mays* L. Int. J. Agric. Res. Rev..

[53] Sodaeizadeh H., Hakimi Maybodi M.H. (2010). Allelopathic effects of *Capparis spinosa, Herttia angustifolia* and *Peganum harmala* on germination and seedling growth of wheat and alfalfa. J. Agricultural Sci. Sustainable Production..

[54] Azwanida N.N. (2015). A review on the extraction methods use in medicinal plants, principle, strength and limitation. Med. Aromatic Plants.

[55] Handa S.S., Khanuja S.P.S., Longo G., Rakesh D.D. (2008).

[56] Rao N.K., Hanson J., Dulloo M.E., Ghosh K., Nowell D., Larinde M. (2006).

[57] Coolbear P., Francis A., Grierson D. (1984). The effect of low temperature pre-sowing treatment on the germination performance and membrane integrity of tomato seeds. J. Exp. Bot..

[58] Ellis R.H., Roberts E.H. (1981). The quantification of ageing and survival in Orthodox seeds. Seed Sci. Technol..

[59] Bewley J.D., Black M. (1994).

[60] Maguire J.D. (1962). Speed of germination—aid in selection and evaluation for seedling emergence and vigor. Crop Sci..

[61] Therneau T.M. (2024). A package for survival analysis in R. R package version.

[62] Kassambara A., Kosinski M., Biecek P., Fabian S. (2025). Survminer: drawing survival curves using 'ggplot2*'*. R package version 0.5.1.

[63] Parvar M., Ahmadi A., Sabeti P. (2010). Allelopathic effects of various plant extracts on the growth of wild mustard and wild oats. Weed Science Journal.

[64] Farhoudi R., Saeedipour S., Sadeghi M. (2015). Allelopathic potential of wheat and barley cultivars on canola germination and seedling growth. Not. Sci. Biol..

[65] Cao J., Dong Z., Zhao H., Duan S., Cao X., Liu H., Yang Z. (2020). Allelopathic effect of rhubarb extracts on the growth of *microcystis aeruginosa*. Water Sci. Technol..

[66] Barmaki M. (2019). Inhibitory effects of allelopathic substances on plant growth. J. Plant Growth Regul..

[67] Jalal A., de Oliveira Junior J.C., Ribeiro J.S., Fernandes G.C., Mariano G.G., Trindade V.D.R., dos Reis A.R. (2021). Hormesis in plants: physiological and biochemical responses. Ecotoxicol. Environ. Saf..

[68] Viator R.P., Johnson R.M., Grimm C.C., Richard E.P. (2006). Allelopathic, autotoxic, and hormetic effects of postharvest sugarcane residue. Agron. J..

[69] Wang C., Liu J., Xiao H., Zhou J., Du D. (2017). Nitrogen deposition influences the allelopathic effect of an invasive plant on the reproduction of a native plant: *solidago canadensis* versus *Pterocypsela laciniata*. Pol. J. Ecol..

[70] Inderjit, Duke S.O. (2003). Ecophysiological aspects of allelopathy. Planta.

[71] Mushtaq W., Fauconnier M.L. (2024). Phenolic profiling unravelling allelopathic encounters in agroecology. Plant Stress.

[72] Weir T.L., Park S.W., Vivanco J.M. (2004). Biochemical and physiological mechanisms mediated by allelochemicals. Curr. Opin. Plant Biol..

[73] Hegazy A.K., Fadl-Allah E.M. (1995). Inhibition of seed germination and seedling growth by *Cleome droserifolia* and allelopathic effect on rhizosphere fungi in Egypt. J. Arid Environ..

[74] Chou C.H., Reigosa M.J., Pedrol N., González L. (2006). Allelopathy: a Physiological Process with Ecological Implications.

[75] Rice E.L. (2012).

[76] Nishida N., Tamotsu S., Nagata N., Saito C., Sakai A. (2005). Allelopathic effects of volatile monoterpenoids produced by *salvia leucophylla*: inhibition of cell proliferation and DNA synthesis in the root apical meristem of *Brassica campestris* seedlings. J. Chem. Ecol..

[77] Cheng F., Cheng Z. (2015). Research progress on the use of plant allelopathy in agriculture and the physiological and ecological mechanisms of allelopathy. Front. Plant Sci..

[78] Einhellig F.A. (1995). Mechanisms of action of allelochemicals. Agron. J..

[79] Chou C.H. (1999). Roles of allelopathy in plant biodiversity and sustainable agriculture. Crit. Rev. Plant Sci..

[80] Reigosa M.J., Pedrol N., González L. (2006). Allelopathy: a Physiological Process with Ecological Implications.

[81] Inderjit, Callaway R.M. (2003). Experimental designs for the study of allelopathy. Plant Soil.

[82] Weston L.A., Duke S.O. (2003). Weed and crop allelopathy. Crit. Rev. Plant Sci..

[83] Öztürk M., Aydoğmuş-Öztürk F., Duru M.E., Topçu G. (2007). Antioxidant activity of stem and root extracts of rhubarb (*Rheum ribes*): an edible medicinal plant. Food Chem..

[84] Binici H.İ. (2025). Antioxidant and bioactive potential of *Rheum ribes* L. flowers: a comprehensive study on secondary metabolites and volatile compounds. J. Agric. Sci..

[85] Erofeeva E.A. (2022). Hormesis in plants: its common occurrence across stresses. Current Opinion. Toxicol..

[86] Patel M., Das S., Yadav S., Srivastava R., Singh R. (2022). Role of cytokinins in seed development in pulses and oilseed crops: current status and future perspective. Front. Genet..

